# Supplementary material for: Integrative Meta-Analysis of Huntington’s Disease Transcriptome Landscape
Source: Genes (Basel). 2022 Dec 16;13(12):2385. doi: 10.3390/genes13122385 (PMC9777612; doi:10.3390/genes13122385)
Supplement: Supplementary file 1 [file genes-13-02385-s001.zip › genes-2043060-supplementary.pdf]

Supplementary Information

Integrative Meta- analysis of Huntington's disease transcriptome landscape

Nela Pragathi Sneha[1], S. Akila Parvathy Dharshini[1], Y-h. Taguchi[2] and

M. Michael Gromiha[1,*]

[1]Department of Biotechnology, Bhupat and Jyoti Mehta School of Biosciences,

Indian Institute of Technology Madras, Chennai 600036, Tamilnadu, India.

[2]Department of Physics, Chuo University, Kasuga, Bunkyo-ku, Tokyo 112-8551, Japan.

---

[*]corresponding author

Tel: +91-2257-4138

Fax: +91-2257-4102

E-mail: gromiha@iitm.ac.in

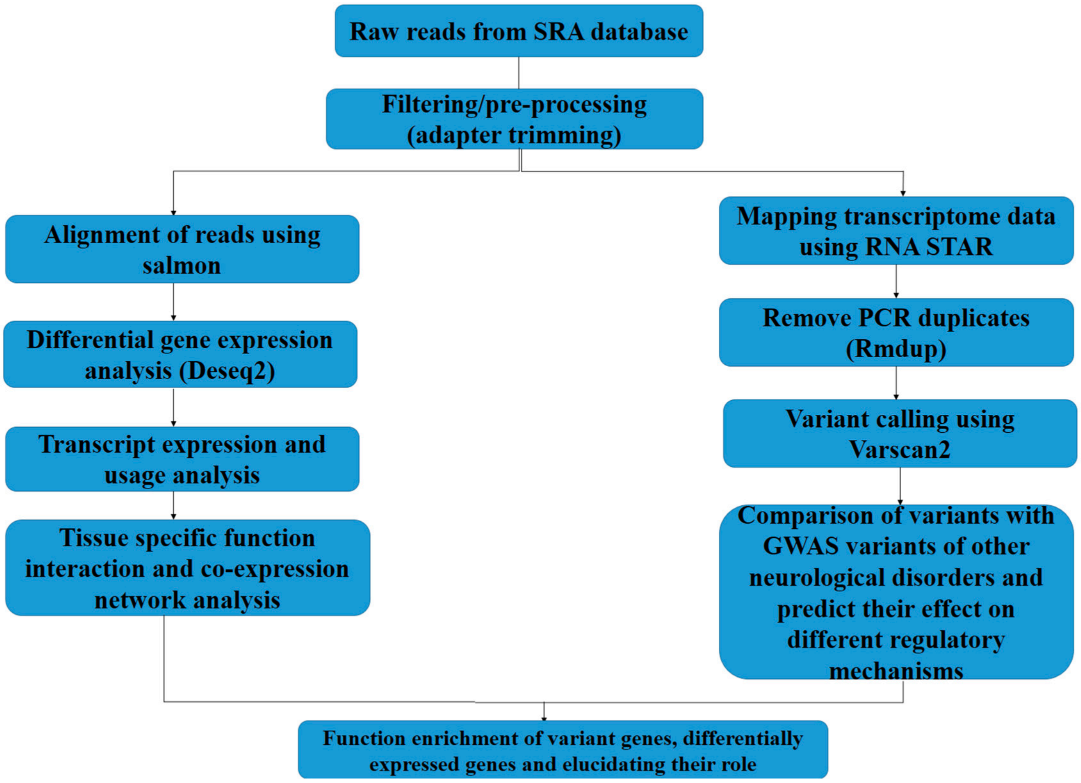

Supplementary Figure S1: Protocol followed for variant calling, differential gene expression and network analysis of RNA-Seq Huntington's data

Supplementary Table S1: Dataset information along with uniquely mapped reads rate(in percentage)

DATASET	SAMPLE	GRADE	UNIQUELY MAPPED READS(%)
SRR1747143	Control	NA	87.5
SRR1747144	Control	NA	81.42
SRR1747145	Control	NA	87.3
SRR1747146	Control	NA	88.6
SRR1747147	Control	NA	80.36
SRR1747148	Control	NA	87.32
SRR1747149	Control	NA	86.59
SRR1747150	Control	NA	85.75
SRR1747151	Control	NA	84.4
SRR1747152	Control	NA	85.59
SRR1747153	Control	NA	81.53
SRR1747154	Control	NA	85.45
SRR1747155	Control	NA	86.82
SRR1747156	Control	NA	82.54
SRR1747157	Control	NA	83.48
SRR1747158	Control	NA	88.37
SRR1747159	Control	NA	81.83
SRR1747160	Control	NA	86.66
SRR1747161	Control	NA	87.29
SRR1747162	Control	NA	83.22
SRR1747163	Control	NA	79.68
SRR1747164	Control	NA	83.53
SRR1747165	Control	NA	88.16
SRR1747166	Control	NA	80.86
SRR1747167	Control	NA	82.36
SRR1747168	Control	NA	93.34
SRR1747169	Control	NA	92.99
SRR1747170	Control	NA	93.44
SRR1747171	Control	NA	92.36
SRR1747172	Control	NA	92.77
SRR1747173	Control	NA	92.91
SRR1747174	Control	NA	92.34
SRR1747175	Control	NA	93.17
SRR1747176	Control	NA	93.76
SRR1747177	Control	NA	90.74
SRR1747178	Control	NA	92.11
SRR1747179	Control	NA	92.68
SRR1747180	Control	NA	88.81
SRR1747181	Control	NA	93.53
SRR1747182	Control	NA	92.94
SRR1747183	Control	NA	92.6
SRR1747184	Control	NA	93.03

SRR1747185	Control	NA	93.09
SRR1747186	Control	NA	94.06
SRR1747187	Control	NA	94.26
SRR1747188	Control	NA	93.31
SRR1747189	Control	NA	93.55
SRR1747190	Control	NA	94.32
SRR1747191	Control	NA	93.68
SRR1747192	HD	Grade 3	92.79
SRR1747193	HD	Grade 3	93.15
SRR1747194	HD	Grade 3	92.79
SRR1747195	HD	Grade 4	93.86
SRR1747196	HD	Grade 4	92.93
SRR1747197	HD	Grade 3	93.21
SRR1747198	HD	Grade 3	92.99
SRR1747199	HD	Grade 3	89.59
SRR1747200	HD	Grade 3	94.46
SRR1747201	HD	Grade 3	92.99
SRR1747202	HD	Grade 3	93.3
SRR1747203	HD	Grade 3	93.84
SRR1747204	HD	Grade 4	93.31
SRR1747205	HD	Grade 3	93.64
SRR1747206	HD	Grade 3	93.4
SRR1747207	HD	Grade 4	93.3
SRR1747208	HD	Grade 3	93.96
SRR1747209	HD	Grade 4	94.32
SRR1747210	HD	Grade 3	93.68
SRR1747211	HD	Grade 4	93.37

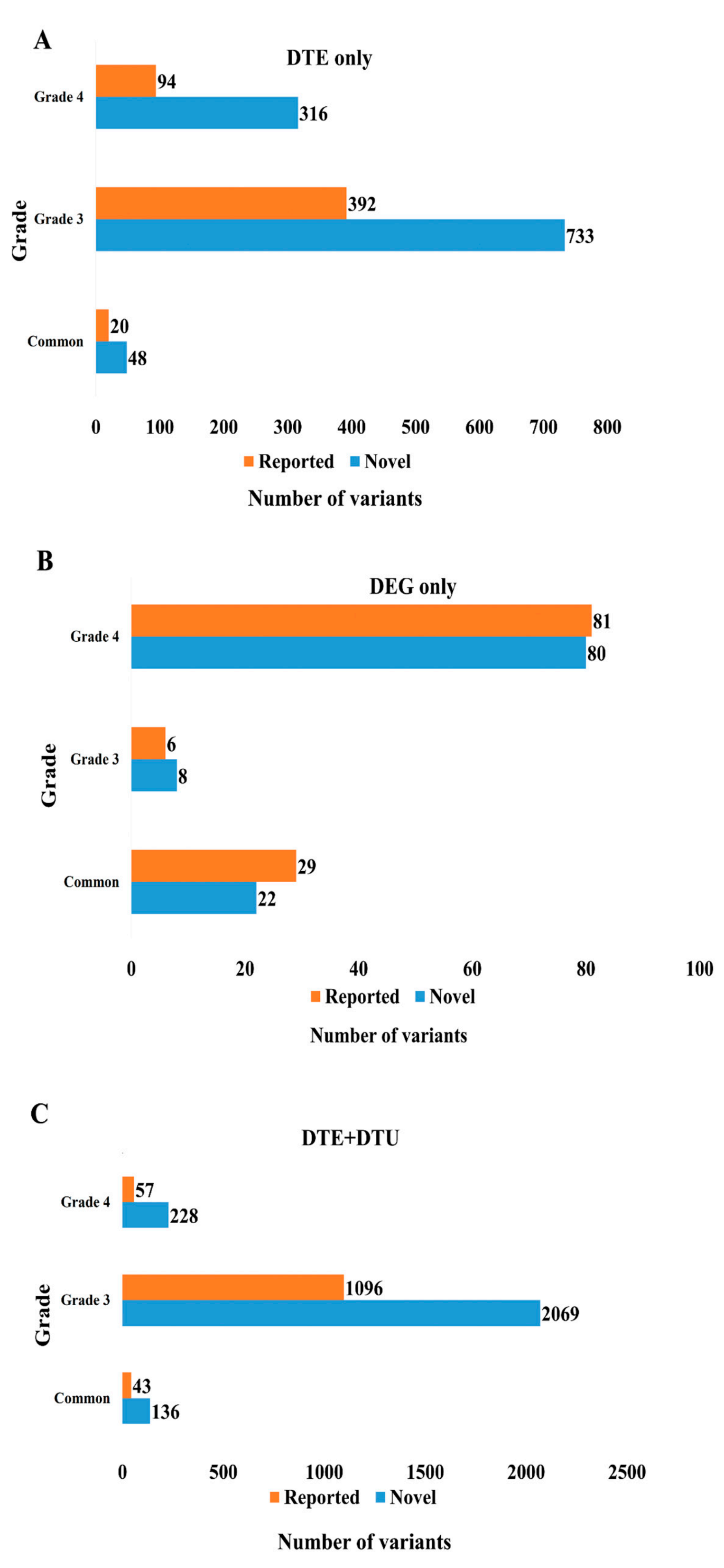

Supplementary Figure S2: Number of variants matching with differential gene expression and transcript expression. **A:** number of variants that are only involved in differential transcript expression. **B:** number of variants which are matching with differentially expressed genes. **C:** number of variants that are involved in both differential transcript usage and expression.

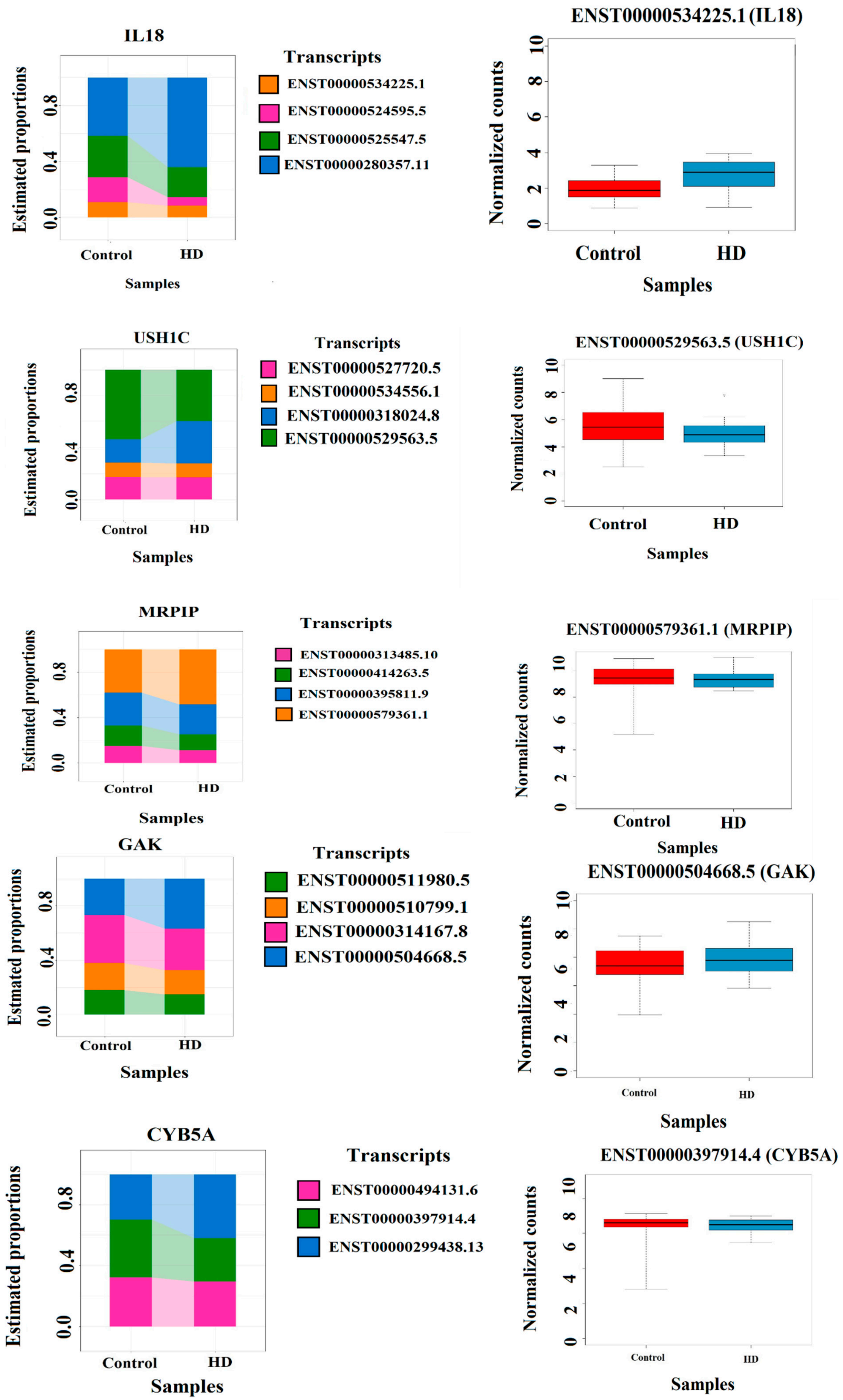

IL18
Estimated proportions
0.0
0.4
0.8
Control
HD
Samples
Transcripts
ENST00000534225.1
ENST00000524595.5
ENST00000525547.5
ENST00000280357.11
ENST00000534225.1 (IL18)
Normalized counts
0
2
4
6
8
10
Control
HD
Samples
USH1C
Estimated proportions
0.0
0.4
0.8
Control
HD
Samples
Transcripts
ENST00000527720.5
ENST00000534556.1
ENST00000318024.8
ENST00000529563.5
ENST00000529563.5 (USH1C)
Normalized counts
0
2
4
6
8
10
Control
HD
Samples
MRPIP
Estimated proportions
0.0
0.4
0.8
Control
HD
Samples
Transcripts
ENST00000313485.10
ENST00000414263.5
ENST00000395811.9
ENST00000579361.1
ENST00000579361.1 (MRPIP)
Normalized counts
0
2
4
6
8
10
Control
HD
Samples
GAK
Estmated proportions
0.0
0.4
0.8
Control
HD
Samples
Transcripts
ENST00000511980.5
ENST00000510799.1
ENST00000314167.8
ENST00000504668.5
ENST00000504668.5 (GAK)
Normalized counts
0
2
4
6
8
10
Control
HD
Samples
CYB5A
Estimated proportions
0.0
0.4
0.8
Control
HD
Samples
Transcripts
ENST00000494131.6
ENST00000397914.4
ENST00000299438.13
ENST00000397914.4 (CYB5A)
Normalized counts
0
2
4
6
8
10
Control
HD
Samples

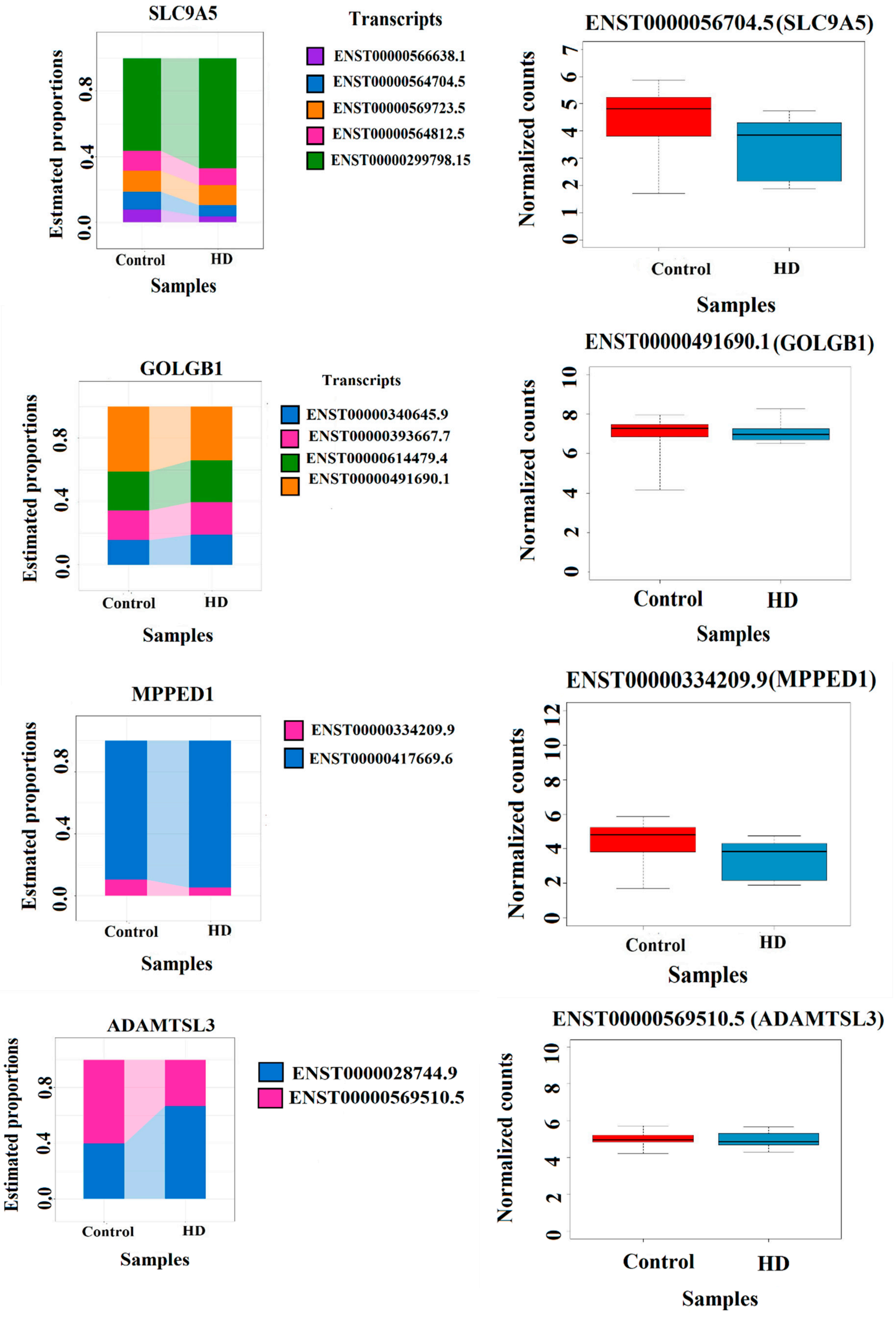

Supplementary Figure S3: Transcript expression and proportion plots for grade 3 and grade 4 variant associated genes. Left: transcript proportion in control and HD. Right: transcript expression plot for a specific transcript.

Supplementary Table S2: Differentially expressed genes of grade 3

GENE	log2fc	p-value	Expression	Reported/Novel
HOXC6	4.79248	7.52E-09	UP	Reported
PITX2	4.305306	2.49E-06	UP	Reported
TEX43	2.810228	7.87E-08	UP	novel
FTH1P15	-2.09352	0.000271	Down	novel
MT-TS1	-4.20254	3.74E-07	Down	Novel
MT-TF	-4.08934	3.31E-07	Down	Novel
MT-TL2	-3.798	1.75E-06	Down	Novel
MT-TW	-2.67014	0.000492	Down	Novel
MT-TD	-3.15898	9.49E-05	Down	Novel
MT-TG	-2.99676	2.07E-05	Down	Novel
MT-TR	-2.74496	0.000134	Down	Novel
CAP1P1	-2.40542	0.000281	Down	novel
MT-TV	-3.23081	0.000107	Down	Novel
MIR663A	-4.00095	6.98E-06	Down	novel
RPL24P2	-2.59167	0.001364	Down	novel
TMEM88B	-3.32378	5.11E-06	Down	Novel
MT-TL1	-3.7368	2.07E-05	Down	Novel
MT-TK	-3.15399	6.59E-05	Down	Novel
MT-TM	-2.893	0.000578	Down	Novel
MTRNR2L8	-4.07728	4.71E-05	Down	Novel
MT-TH	-2.91429	0.000263	Down	Novel
MT-TN	-2.66737	0.000316	Down	Novel
MT-TA	-2.17954	0.00204	Down	novel
ZNRF2P1	-3.78985	0.002167	Down	novel
GACAT3	-4.48829	6.85E-05	Down	Novel
HOXA10	4.934067	3.76E-21	UP	Reported
CXCR2	3.021769	3.53E-12	UP	Reported
B2M	2.583597	9.09E-14	UP	Reported
CLIC6	2.837127	6.04E-12	UP	Novel
HOXC6	4.79248	7.52E-09	UP	Reported
INSRR	2.761596	1.65E-05	UP	Novel
MNX1	2.949218	6.13E-08	UP	REPORTED
PITX2	4.305306	2.49E-06	UP	Reported
TDRD15	3.807996	0.000146	UP	novel
TEX43	2.810228	7.87E-08	UP	novel

Supplementary Table S3: Differentially expressed genes of grade 4

gene	log2FC	p-value	expression	Novel/reported
CD177	5.945288	4.45E-16	UP	Novel
SLC47A1	3.339228	5.93E-19	UP	Reported
UPK1B	5.281816	3.29E-14	UP	Novel
F13A1	4.186481	5.23E-16	UP	Reported
LINC01133	3.681797	7.31E-15	UP	Novel
H19	3.670979	3.3E-14	UP	Novel
GAS1	2.379779	1.01E-14	UP	Reported
LINC01638	4.477892	6.14E-13	UP	Novel
TMEM30B	3.913632	2.14E-13	UP	Reported
THSD4	2.377644	1.19E-12	UP	Novel
VNN2	3.5514	1.1E-11	UP	Reported
SLC22A6	3.245746	1.64E-11	UP	Novel
TBX15	3.067255	2.63E-11	UP	Novel
IGHG3	5.205675	2.81E-08	UP	Novel
OMD	2.739056	4.68E-11	UP	Novel
UGT3A2	4.474519	2.04E-09	UP	Novel
SLC26A2	2.827773	2.06E-10	UP	Reported
LRRN4CL	2.301129	1.33E-10	UP	Novel
IL18	2.484263	3.33E-10	UP	Reported
FGL2	2.923651	8.35E-10	UP	Novel
CLIC3	2.858753	7.52E-10	UP	Reported
CPXM2	2.553845	6.07E-10	UP	Novel
FOXC2	4.048598	1.53E-08	UP	Reported
SFRP2	2.944392	1.32E-09	UP	Novel
DEFB1	5.427186	5.94E-06	UP	Reported
SPTLC3	2.3523	8.92E-10	UP	Reported
CRYGN	2.713751	3.59E-09	UP	Novel
MRAP	4.065933	7.1E-08	UP	Novel
MARCO	4.493875	7.64E-07	UP	Reported
OTOG	2.643243	4.67E-09	UP	Novel
S100A4	2.902545	1.05E-08	UP	Reported
CXorf49	6.450403	5.81E-05	UP	Novel
STRA6	2.437824	1.21E-08	UP	Reported
OSR1	2.73528	3.86E-08	UP	Novel
SLC38A4	3.137126	9.53E-08	UP	Reported
LYVE1	2.797669	6.53E-08	UP	Reported
IGKV3-20	6.296459	0.000953	UP	Novel
WNT6	2.806684	9.5E-08	UP	Novel
OLFML2A	2.515984	7.7E-08	UP	Reported
MT1G	2.495813	9.68E-08	UP	Reported
PRRX2	3.350885	4.66E-07	UP	Novel
ANXA2	2.566377	1.37E-07	UP	Reported
SLC5A5	2.515898	1.95E-07	UP	Reported
CAPN6	4.484365	2E-05	UP	Reported
CCR2	3.846334	2.05E-06	UP	Reported

GZMK	4.401841	2.39E-05	UP	Reported
B3GNT8	2.621938	4.98E-07	UP	Novel
GPR183	3.077225	1.38E-06	UP	Reported
CD48	3.120039	1.35E-06	UP	Reported
KRT18	3.431178	4.09E-06	UP	Reported
IGLC2	4.913089	0.000448	UP	Novel
PROKR1	2.856523	1.16E-06	UP	Novel
COL2A1	2.649043	1.29E-06	UP	Reported
EGFLAM	2.959986	2.77E-06	UP	Novel
KRT19	2.746069	2.65E-06	UP	Novel
ECEL1	2.618588	2.41E-06	UP	Reported
SIX6	4.522254	0.000309	UP	Novel
IGHG2	3.422468	1.74E-05	UP	Novel
EDN2	4.510519	0.000225	UP	reported
MS4A4A	2.720249	7.51E-06	UP	Reported
FOXI2	3.367004	2.35E-05	UP	Novel
RGS1	3.098292	2.32E-05	UP	Novel
LINC02692	3.675713	7.03E-05	UP	Novel
FCER1A	3.690188	0.000215	UP	Reported
NPHS1	-2.50879	1.49E-12	Down	Novel
USP17L2	-4.61073	1.3E-06	Down	Novel
MIR149	-4.35341	6.99E-06	Down	Novel
MRC1	3.333906	3.79E-11	UP	Novel
PDGFD	2.361297	9.63E-09	UP	Reported
TLR8	3.108346	1.83E-06	UP	Reported
UQCRFS1P1	-3.36458	4.82E-08	Down	Novel
